# Comparison of four commercial RT‐PCR diagnostic kits for COVID‐19 in China

**DOI:** 10.1002/jcla.23605

**Published:** 2020-12-15

**Authors:** Lingyu Shen, Shujuan Cui, Daitao Zhang, Changying Lin, Lijuan Chen, Quanyi Wang

**Affiliations:** ^1^ Institute of Infectious Diseases and Endemic Diseases Prevention and Control, Beijing Municipal Center for Disease Prevention and Control Beijing Municipal Research Center for Preventive Medicine Beijing P. R. China

**Keywords:** COVID‐19, RT‐PCR, sensitivity, specificity

## Abstract

We compared the sensitivity and specificity of four commercial coronavirus disease (COVID‐19) diagnostic kits using real‐time reverse transcription–polymerase chain reaction (RT‐PCR). Kits I‐IV approved by the State Drug Administration of China were selected, and the detection targets were ORF1ab gene and N gene. Specificity was evaluated by detecting other respiratory viruses. The sensitivity and batch effect of each kit were evaluated by testing 10‐fold dilutions of RNA. Clinical application was verified by testing nasopharyngeal swab and sputum specimens from COVID‐19 patients. Among the 78 cases infected by other respiratory viruses, no amplification curve was observed using these four COVID‐19 RT‐PCR kits. The minimum detection limits of kits I‐IV were 10^−6^, 10^−5^, 10^−5^, and 10^−6^ dilutions, respectively, and concentrations were 10 copies/mL (10^−5^ dilution) and 1 copies/mL (10^−6^ dilution). The sensitivities of kits I‐IV detected using 142 nasopharyngeal swab specimens from COVID‐19 patients were 91.55%, 81.69%, 80.28%, and 90.85%, respectively, while they were 92.68%, 85.37%, 82.93%, and 93.90%, respectively, for the 82 sputum samples. The specificity of each kit was 100.00% (77/77). The total expected detection rate using sputum samples was 88.59% (691/780) higher than 86.15% (672/780) of nasopharyngeal swabs. Comparison of nasopharyngeal swab and sputum samples from the same COVID‐19 patient led to the detection of ORF1ab and N genes in 16 (100%) sputum samples; only ORF1ab and N genes were detected in 12 (75%) and 14 (87.5%) nasopharyngeal swab specimens, respectively. In conclusion, comparison of commercial COVID‐19 RT‐PCR kits should be performed before using a new batch of such kits in routine diagnostics.

## Graphical abstract

Suspected COVID‐19 cases with low RNA concentration or at the initial stages of the disease should be examined using different COVID‐19 kits or sampling sputum specimens to a feasible extent and comparison of commercial COVID‐19 RT‐PCR kits should be performed prior to using new batches of the kits in routine diagnostics.

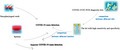

## 1 INTRODUCTION

Severe acute respiratory syndrome coronavirus 2 (SARS‐CoV‐2) emerged among humans during the final months of 2019, causing severe acute respiratory diseases, multiple organ injuries, and fatal outcomes.^1^, ^2^, ^3^, ^4^ The resulting disease, therefore, has been named coronavirus disease (COVID‐19).^1^, ^2^, ^3^ SARS‐CoV‐2 is a human coronavirus (HCoV). HCoVs are enveloped viruses with a single‐stranded, positive‐sense RNA and belong to the order *Nidovirales*.^5^, ^6^ The length of HCoVs is approximately 27‐32 kilobases, and these viruses are divided into seven species, including HCoV‐229E, HCoV‐NL63, HCoV‐OC43, HCoV‐HKU1, SARS‐CoV, MERS‐CoV, and SARS‐CoV‐2.^7^, ^8^

Real‐time reverse transcription–polymerase chain reaction (RT‐PCR) is the most sensitive and specific assay that can provide crucial etiological evidence for COVID‐19 diagnosis.^9^, ^10^ The coronavirus nucleocapsid (N) protein is expressed through the production of subgenomic messenger RNAs, and the number of N proteins markedly exceeds that of genomic RNAs in several stages of the replication cycle.^6^, ^8^, ^11^, ^12^ The RNA‐dependent RNA polymerase, called ORF1ab, is the main region for virus replication and transcription.^6^, ^8^, ^11^, ^12^ Therefore, both ORF1ab and N genes are the crucial targets used for RT‐PCR‐based SARS‐CoV‐2 detection.^13^, ^14^

Recently, the efficacy of RT‐PCR for COVID‐19 diagnosis has been questioned.^15^ Although several COVID‐19 RT‐PCR diagnostic kits are commercially available, the detection rates of SARS‐CoV‐2 infection have been unsatisfactory, and several cases have been detected following negative detection results obtained from repeated RT‐PCR laboratory diagnostic tests and COVID‐19 features already observed on computed tomography images.^16^, ^17^, ^18^, ^19^, ^20^

Currently, there is no better diagnostic method for COVID‐19 than RT‐PCR.^21^ The most significant steps for SARS‐CoV‐2 detection in a RT‐PCR diagnostic laboratory are to identify and use RT‐PCR kits with high sensitivity and specificity.^16^, ^17^, ^18^, ^19^, ^20^ Comparisons of sensitivity and specificity among different commercial RT‐PCR diagnostic kits are still limited. Moreover, there is a dearth of information on the comparison methods. Therefore, this study aimed to compare the sensitivity and specificity among four commercial COVID‐19 RT‐PCR diagnostic kits from different manufacturers and suggest comparison methods that may be employed to identify efficient kits for routine diagnostics.

## 2 METHODS

### 2.1 Selection of kit

Four commercially available COVID‐19 RT‐PCR diagnostic kits (kits I, II, III, and IV) from different manufacturers certified by the State Food and Drug Administration in China were selected for comparison in this study. The manufacturers included Beijing Applied Biological Technologies Co., Ltd; Beijing Kinghawk Pharmaceutical Co., Ltd; Beijing NaGene Diagnosis Reagent Co., Ltd; and Coyote Bioscience Co., Ltd (listed alphabetically). All the kits were suitable for ABI 7500 real‐time PCR system, and the detection targets were ORF1ab gene, N gene, and ribonucleoprotein (RNP).

### 2.2 Clinical specimens

Clinical specimens, including nasopharyngeal swab and sputum specimens, were collected from confirmed or excluded COVID‐19 patients experiencing acute respiratory tract infection. The criteria for confirmed COVID‐19 cases included the following: (a) already‐confirmed cases of COVID‐19; (b) nasopharyngeal swab and sputum specimens positive for SARS‐CoV‐2 nucleic acids upon detection by RT‐PCR tests performed at the Beijing Center for Disease Prevention and Control (BJCDC); and (c) cases that obtained a fraction of the RNA sequence of SARS‐CoV‐2 by PCR amplifications and gene sequencing. Exclusion of cases was based on both clinical evidence and RT‐PCR results. This study was approved by the Ethics Committee at BJCDC.

### 2.3 Nucleic acid extraction

Total nucleic acids (RNA and DNA) were extracted from the clinical specimens using Thermo Scientific^™^ KingFisher^™^ Flex Magnetic Particle Processors (cat no. KFR‐805496; Thermo Fisher: Waltham, MA). Approximately 60 µL of total nucleic acid eluates was recovered in nuclease‐free tubes and tested immediately.

### 2.4 Specificity assessment

Nasopharyngeal swab specimens containing other respiratory viruses, including influenza virus A, influenza virus B, respiratory syncytial virus, parainfluenza virus, human adenovirus, human rhinovirus, other HCoVs (NL63, OC43, 229E, and HKU1), human metapneumovirus, and human bocavirus, were also collected to identify the specificity of the assessed kits. A commercial RT‐PCR kit (cat. no. CN12‐33‐100; Jiangsu Uninovo Biological Technology Co. Ltd., Jiangsu, China) was used to detect these viruses.

### 2.5 Sensitivity assessment

Tenfold serial dilutions (1:10 to 1:10^6^) of nucleic acid eluates from a COVID‐19 patient (*C_t_* = 17) were prepared in duplicate. The concentrations of nucleic acid eluates (10^−1^ to 10^−6^ dilutions) were detected by digital PCR. Three batches (batches A, B, and C) of the four kits were selected to evaluate sensitivity and batch effects. Sensitivity was evaluated by testing all serial dilutions with the kits. Further, batch effects were evaluated using linear regression analyses. *R* ^2^ values of the linear regression analyses were obtained using SPSS software, version 20.0: IBM, Amonk, NY. All charts were made using Origin software, version 9.0: OriginLab, Northampton, Ma.

### 2.6 Clinical application

Clinical application was verified by testing nasopharyngeal swab and sputum specimens of confirmed or excluded COVID‐19 cases using the four RT‐PCR kits. Head‐to‐head comparison of SARS‐CoV‐2 detection among nasopharyngeal swab and sputum samples from the same patient was performed to show whether different types of specimens from the same patient would affect the testing capabilities of the kits. Simultaneous detection of both ORF1ab and N genes indicated a positive COVID‐19 test. A case with single positive result of ORF1ab or N gene was diagnosed as a suspected case. RNP positivity alone was diagnosed as negative. The specific cutoff values of the four kits are listed in Table 1. For suspected and inconsistent samples, we repeated the RT‐PCR test according to the manufacturers’ instructions of the kits, and the final results of the repeated tests were used. Kappa value, sensitivity, and specificity were calculated using SPSS software, version 20.0. The Kappa values corresponding to weak, moderate, high, and strong consistencies were >0.20 to ≤0.40, >0.40 to ≤0.60, >0.60 to ≤0.80, and >0.80 to ≤1.00, respectively.

**TABLE 1:** Specific standards of the four COVID‐19 RT‐PCR diagnostic kits

Kits	Standard		
	Positive	Negative	Suspicion
I	0 < CT ≤ 38.00	NO CT or CT ≥ 40.00	38.00 < CT < 40.00
II	0 < CT ≤ 38.00	NO CT or CT> 40.00	38.00 < CT ≤ 40.00
III	0 < CT < 37.00	NO CT or CT ≥ 40.00	37.00 ≤ CT < 40.00
IV	0 < CT ≤ 40.00	NO CT or CT ≥ 40.00	—

Abbreviations: COVID‐19, coronavirus disease‐19; CT, computed tomography; RT‐PCR, real‐time reverse transcription–polymerase chain reaction.

## 3 RESULTS

### 3.1 Specificity assessment

Samples from 78 patients diagnosed with acute respiratory tract infections caused by other respiratory viruses apart from SARS‐CoV‐2 were collected, including 20 cases of influenza virus A, 20 cases of influenza virus B, 20 cases of HCoVs (5 cases each of NL63, OC43, 229E, and HKU1), 3 cases of respiratory syncytial virus, 3 cases of parainfluenza virus, 3 cases of human adenovirus, 3 cases of human rhinovirus, 3 cases of human metapneumovirus, and 3 cases of human bocavirus. No amplification curve was observed for the samples tested using the four RT‐PCR kits.

### 3.2 Sensitivity assessment

**FIGURE 1:**
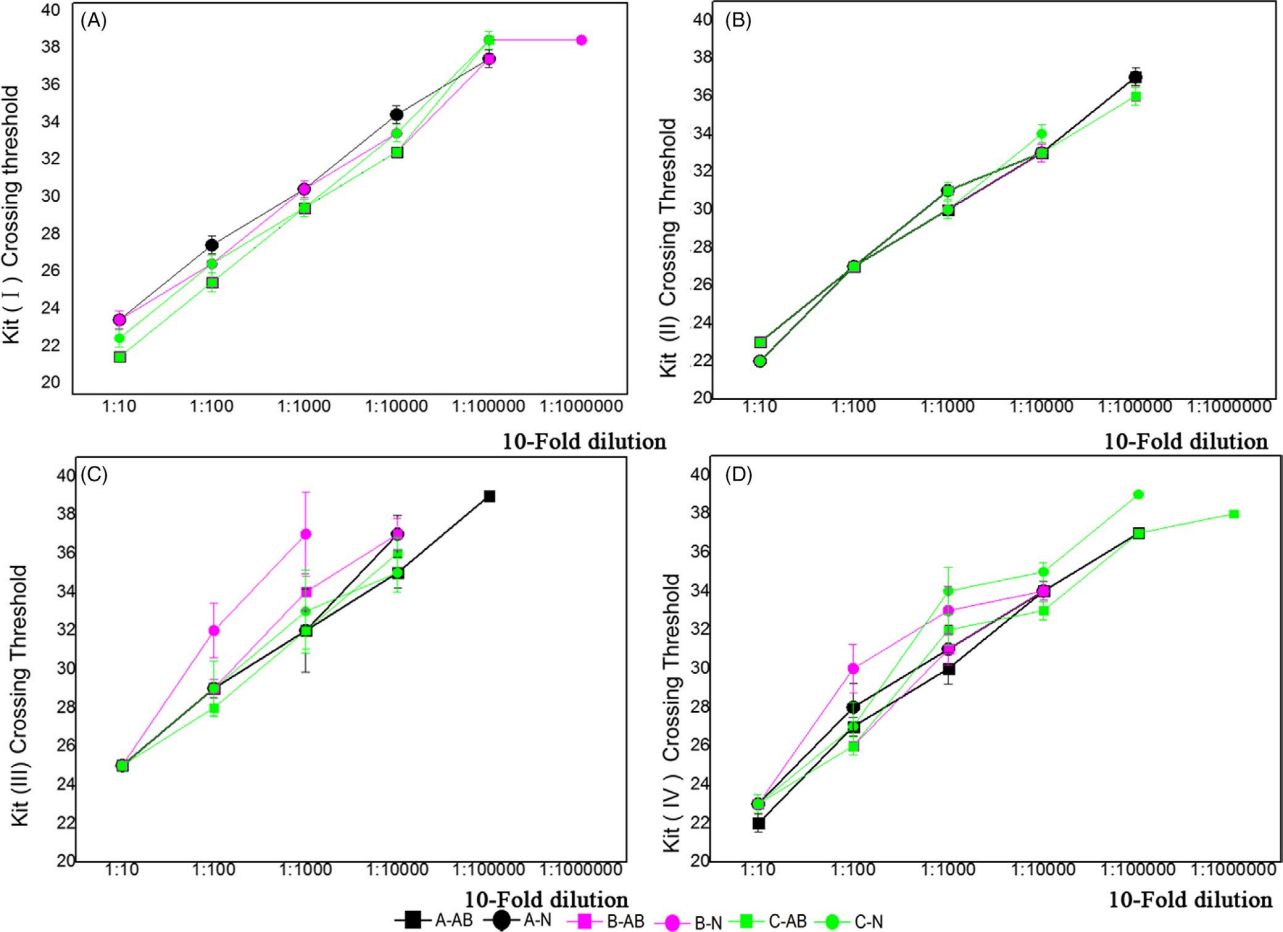
Sensitivities and batch effects of batches A, B, and C of four commercial kits for severe acute respiratory syndrome coronavirus 2 detection. Sensitivities and batch effects were evaluated by testing serial 10‐fold dilutions of RNA samples from coronavirus disease‐19 patients using three batches of the kits. ORF1ab gene targets are marked with square, N gene targets with circle. Lines representing batch A are indicated in black, batch B in purple, and batch C in green. (a) Sensitivities and batch effects of batches A, B, and C of kit I; (b) sensitivities and batch effects of batches A, B, and C of kit II; (c) sensitivities and batch effects of batches A, B, and C of kit III; (d) sensitivities and batch effects of batches A, B, and C of kit IV

Ten fold serial dilutions of nucleic acid eluates of the COVID‐19 case were tested in duplicate to evaluate the sensitivity of the kits. The concentrations of the nucleic acid eluates (10^−1^ to 10^−6^ dilutions) were 27 230 copies/mL, 5399 copies/mL, 1395 copies/mL, 437 copies/mL, 10 copies/mL, and 1 copies/mL, respectively, as detected by digital PCR. The minimum detection limits of the ORF1ab gene and N gene targets of kit I (10^−5^ dilution of ORF1ab gene and 10^−6^ dilution of N gene), kit II (10^−5^ dilution of ORF1ab gene and 10^−5^ dilution of N gene), kit III (10^−5^ dilution of ORF1ab gene and 10^−5^ dilution of N gene), and kit IV (10^−6^ dilution of ORF1ab gene, and 10^−5^ dilution of N gene) are shown in Figure 1. Not all gene targets of kits I to IV could be detected in high dilutions with low RNA concentration in most cases.

Batch effect was evaluated by testing the detection difference between three batches of the assessed kits, and the data were analyzed by linear correlation analysis using SPSS software, version 20.0. The *R* ^2^ values of the tested batches of the same kit showed slight differences. The *R* ^2^ mean values were as follows: (a) kit I: ORF1ab gene, 0.9933; and N gene, 0.9869; (b) kit II: ORF1ab gene, 0.9696; and N gene, 0.9848; (c) kit III: ORF1ab gene, 0.9548; and N gene, 0.9707; and (d) kit IV: ORF1ab gene, 0.9791, and N gene. Additionally, for the four kits, the standard deviations of *R* ^2^ values were as follows: ORF1ab gene, 0.0039, 0.0259, 0.051, and 0.0199; N gene, 0.0146, 0.053, 0.0088, and 0.0347, respectively.

### 3.3 Clinical application

A total of 301 nasopharyngeal swab and sputum specimens were collected between January and March 30, 2020, including 224 and 77 specimens of confirmed and excluded cases, respectively. Among kits I to IV, the positive detection rates of the specimens from the confirmed COVID‐19 patients were 91.96% (206/224), 83.04% (186/224), 81.25% (182/224), and 91.96% (206/224), respectively, and the negative detection rates of the specimens from the excluded COVID‐19 patients were 100.00% (77/77).

### 3.4 Clinical application of nasopharyngeal swab specimens

The types and corresponding patients of the 189 specimens are shown in Table 2. The positive detection numbers of 142 nasopharyngeal swab specimens from the confirmed COVID‐19 patients using kits I to IV were 130, 116, 114, and 129, respectively, and the sensitivities were 91.55% (95% CI*:* 85.70‐95.56), 81.69% (95% CI*:* 74.33‐87.68), 80.28% (95% CI*:* 72.78‐86.48), and 90.85% (95% CI*:* 84.85‐95.04), respectively. The negative detection numbers of 47 nasopharyngeal swab specimens from the excluded COVID‐19 patients using kits I to IV were 47, and the specificities of kits I to IV were all 100.00% (95% CI: 92.45‐100.00).

**TABLE 2:** Detection of targets in the nasopharyngeal swab specimens from COVID‐19 patients using four commercial kits

Kits		Diagnosis of COVID‐19		Total	Sensitivity	Specificity	AUC	*κ*
Manufacturers	Detection	Confirmed	Excluded		(95% CI)	(95% CI)	(95% CI)	(95% CI)
I	+	130	0	130	91.55% (85.70‐95.56)	100.00% (92.45‐100.00)	0.96 (0.92‐0.98)	0.84 (0.80‐0.89)
	−	12	47	59				
	Total	142	47	189				
II	+	116	0	116	81.69% (74.33‐87.68)	100.00% (92.45‐100.00)	0.91 (0.86‐0.95)	0.69 (0.64‐0.74)
	−	26	47	73				
	Total	142	47	189				
III	+	114	0	114	80.28% (72.78‐86.48)	100% (92.45‐100.00)	0.90 (0.85‐0.94)	0.67 (0.62‐0.72)
	−	28	47	75				
	Total	142	47	189				
IV	+	129	0	129	90.85% (84.85‐95.04)	100.00% (92.45‐100.00)	0.95 (0.91‐0.98)	0.83 (0.79‐0.88)
	−	13	47	60				
	Total	142	47	189				

Abbreviations: AUC, area under the curve; COVID‐19, coronavirus disease‐19.

The area under the curve values of the four kits for nasopharyngeal swab specimens were all >0.9, including 0.96 (95% CI*:* 0.92‐0.98) for kit I, 0.95 (95% CI*:* 0.91‐0.98) for kit IV, 0.91 (95% CI*:* 0.86‐0.95) for kit II, and 0.90 (95% CI*:* 0.85‐0.94) for kit III. The consistencies of kits I and IV were strong, with *κ* values 0.84 (95% CI*:* 0.80‐0.89) and 0.83 (95% CI*:* 0.79‐0.88), respectively. The consistencies of kits II and III were high, with *κ* values 0.69 (95% CI*:* 0.64‐0.74) and 0.67 (95% CI*:* 0.62‐0.72), respectively.

### 3.5 Clinical application of the sputum specimens

Among the 301 specimens, 112 were sputum samples, including 82 specimens from confirmed COVID‐19 patients and 30 specimens from excluded COVID‐19 patients, as shown in Table 3. The positive detection numbers of the specimens from the confirmed COVID‐19 patients using kits I to IV were 76, 70, 68, and 77, respectively, and the sensitivities were 92.68% (95% CI*:* 84.75‐97.27), 85.37% (95% CI*:* 75.83‐92.20), 82.93% (95% CI*:* 73.02‐90.34), and 93.90% (95% CI*:* 86.34‐97.99), respectively. Further, the detection numbers of the specimens from the excluded COVID‐19 patients using kits I‐IV were all 30, and the specificities of the kits were all 100% (95% CI*:* 88.43‐100.00).

**TABLE 3:** Detection of targets in the sputum specimens from COVID‐19 patients using four commercial kits

Kits		Diagnosis of COVID‐19		Total	Sensitivity	Specificity	AUC	*κ*
Manufacturers	Detection	Confirmed	Excluded		(95% CI)	(95% CI)	(95% CI)	(95% CI)
I	+	76	0	76	92.68% (84.75‐97.27)	100% (88.43‐100.00)	0.96 (0.91‐0.99)	0.87 (0.82‐0.92)
	−	6	30	36				
	Total	82	30	112				
II	+	70	0	70	85.37% (75.83‐92.20)	100% (88.43‐100.00)	0.93 (0.86‐0.97)	0.76 (0.69‐0.82)
	−	12	30	42				
	Total	82	30	112				
III	+	68	0	68	82.93% (73.02‐90.34)	100% (88.43‐100.00)	0.92 (0.85‐0.96)	0.72 (0.66‐0.79)
	−	14	30	44				
	Total	82	30	112				
IV	+	77	0	77	93.90% (86.34‐97.99)	100% (88.43‐100.00)	0.97 (0.92‐0.99)	0.89 (0.85‐0.94)
	−	5	30	35				
	Total	82	30	112				

Abbreviations: AUC, area under the curve; COVID‐19, coronavirus disease‐19.

The area under the curve values of the four kits for sputum specimens were all >0.9, including 0.97 (95% CI*:* 0.92‐0.99) for kit IV, 0.96 (95% CI*:* 0.91‐0.99) for kit I, 0.93 (95% CI*:* 0.86‐0.97) for kit II, and 0.92 (95% CI*:* 0.85‐0.96) for kit III. The consistencies of kits I and IV were strong, with *κ* values 0.87 (95% CI*:* 0.82‐0.92) and 0.89 (95% CI*:* 0.85‐0.94), respectively. Further, the consistencies of kits II and III were high, with *κ* values 0.76 (95% CI*:* 0.69‐0.82) and 0.72 (95% CI*:* 0.66‐0.79), respectively.

### 3.6 Detection rates of sputum and nasopharyngeal swab specimens

Direct standardization was performed using SPSS software, version 20.0, to select sample type between the nasopharyngeal swab and sputum specimens for SARS‐CoV‐2 detection. The expected detection numbers of all the specimens are shown in Table 4, and the total expected detection rate of the sputum specimens was 88.59% (691/780), which was higher than that of the nasopharyngeal swab specimens (86.15%; 672/780).

**TABLE 4:** Expected detection rates in the nasopharyngeal swab and sputum specimens from COVID‐19 patients

Kits	Standard detection number	Nasopharyngeal swab specimens		Sputum specimens	
Manufacturers		Actual detection rate	No. expected detection	Actual detection rate	No. expected detection
I	206	91.55%	188	92.68%	190
II	186	81.69%	151	85.37%	158
III	182	80.28%	146	82.93%	150
IV	206	90.85%	187	93.90%	193
Total	780	—	672	—	691

Abbreviations: COVID‐19, coronavirus disease‐19.

**FIGURE 2:**
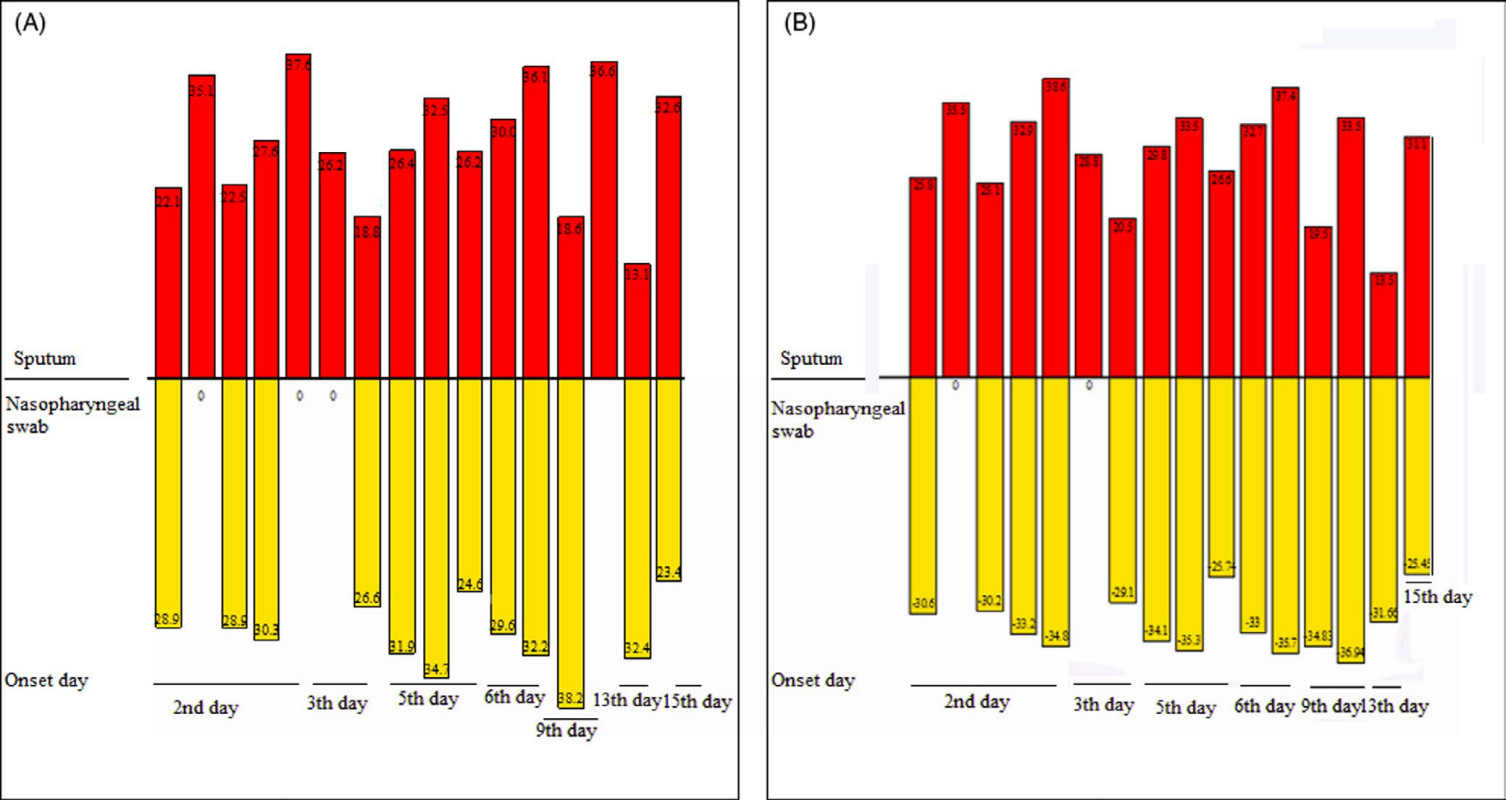
Head‐to‐head comparisons of severe acute respiratory syndrome coronavirus 2 detection among nasopharyngeal swab and sputum specimens from the same patients using kit I. The sputum specimens are indicated in red and nasopharyngeal swab specimens in yellow. A, ORF1ab gene detection in nasopharyngeal swab and sputum specimens; (B) N gene detection in nasopharyngeal swab and sputum specimens

Further, nasopharyngeal swab and sputum specimens were collected from 16 COVID‐19 patients between January and March 30, 2020, and used for head‐to‐head comparisons to show whether different types of specimens from the same patient would affect the testing capabilities of the kits. The clinical information and *C_t_* values of kits I and IV are shown in Table S1. The head‐to‐head comparison of sputum and nasopharyngeal swab specimens collected from the same COVID‐19 patient is shown in Figure 2. It revealed that ORFlab and N genes were detected in all (16; 100%) sputum specimens; only ORFlab and N genes were detected in 12 (75%) and 14 (87.5%) nasopharyngeal swab specimens, respectively. The *C_t_* values of 12 sputum specimens tested for ORFlab and N genes were higher than those of the nasopharyngeal swab specimens.

## 4 DISCUSSION

COVID‐19 outbreaks have become a global public health concern.^10^ Early detection of the disease, quarantine of patients, and diagnosis have been reported to be crucial for controlling its spread, with RT‐PCR being a significant method for detection and diagnosis.^16^, ^22^ The sensitivity of COVID‐19 RT‐PCR diagnostic kits is not only related to the types, sampling, transportation, and preservation of the viral specimens but also to the quality of the kits, which is considered the most important factor.^17^ COVID‐19 RT‐PCR diagnostic kits with high sensitivity and specificity could help reduce the rate of false‐negative detection and significantly improve the identification of COVID‐19 patients.^23^

This study showed that detection rates in the sputum specimens from the lower respiratory tract of COVID‐19 patients using kits I to IV were higher than those in the nasopharyngeal swab specimens from the upper respiratory tract of the patients. Several studies have shown that the sampling quality of specimens obtained from the upper respiratory tract cannot be guaranteed, and specimens with low RNA concentration from the initial stage of COVID‐19 cases also lead to false‐negative detection.^18^, ^19^, ^24^ Detection rates in sputum specimens from the lower respiratory tract are expected to be better than those in nasopharyngeal swab specimens.^18^, ^19^, ^24^ However, patients with weak constitution cannot cough up sputum from the lower respiratory tract and only cough up a small amount of it from the upper respiratory tract, which ultimately leads to false‐negative detection.^18^, ^19^, ^24^

According to the manufacturers’ instructions of the different RT‐PCR kits for COVID‐19 diagnosis available commercially, minimum detection limits are 100‐1000 copies/mL, and a few kits could reach 20 copies/mL.^14^ Although these minimum detection limits are sufficiently low, this study found that the sensitivities of the four assessed kits were slightly different for different targets. N gene and ORF1ab gene targets of SARS‐CoV‐2 could not be concurrently detected in high‐dilution samples containing low RNA concentration. Thus, it is possible that positive cases could be falsely identified to be negative and thereby missed. Thus, we suggest that two or three kits should be used in the attempts to identify COVID‐19 patients to improve the efficacy of the identification process.

Additionally, the results of batch effects among the four kits showed that the abilities to detect the same gene target were slightly different between different batches of the same kit, and overall batch effects were slightly different between the tested commercial kits. Although there might be differences in the raw materials and production lines employed in producing COVID‐19 diagnostic kits, manufacturers should ramp‐up supervision to ensure product quality from batch to batch, which may result in only a slight detection difference within an allowable error range. Further, criteria of comparison should be formulated and comparisons should be made to confirm the sensitivity and specificity of the kits prior to using new batches.

In conclusion, the sensitivities and batch effects of the assessed kits were slightly different for different targets, and sputum specimens were more applicable for SARS‐CoV‐2 detection than nasopharyngeal swab specimens. Therefore, these data suggest that suspected COVID‐19 cases with low RNA concentration or at the initial stages of the disease should be examined using different COVID‐19 kits or sampling sputum specimens to a feasible extent and that comparison of commercial COVID‐19 RT‐PCR kits should be performed prior to using new batches of the kits in routine diagnostics. Additionally, with the increasing number of commercial COVID‐19 kits, it is necessary for researchers to share information such as multi‐center kit comparison methods and the detection abilities of various commercial RT‐PCR diagnostic kits among different specimens.

## Supporting information

Table S1
